# Pseudo-Eutectic of Isodimorphism to Design Biaxially-Oriented Bio-Based PA56/512 with High Strength, Toughness and Barrier Performances

**DOI:** 10.3390/polym16081176

**Published:** 2024-04-22

**Authors:** Diansong Gan, Yuejun Liu, Tianhui Hu, Shuhong Fan, Lingna Cui, Guangkai Liao, Zhenyan Xie, Xiaoyu Zhu, Kejian Yang

**Affiliations:** 1 Key Laboratory of Advanced Packaging Materials and Technology of Hunan Province, School of Packaging and Materials Engineering, Hunan University of Technology, Zhuzhou 412007, China; gands@zztep.cn (D.G.); fs1h1@163.com (S.F.); cuilingna@hut.edu.cn (L.C.); 14158@hut.edu.cn (G.L.); xzy045@foxmail.com (Z.X.); z18363110906@163.com (X.Z.); 2 Zhuzhou Times Engineering Plastics Industrial Co., Ltd., Zhuzhou 412008, China; huth@zztep.cn; 3 School of Chemical Engineering and Light Industry, Guangdong University of Technology, Guangzhou 510006, China

**Keywords:** biaxially-oriented stretching process, bio-based BOPA-56/512, isodimorphism, aggregation structure, mechanical properties

## Abstract

The biaxially-oriented PA56/512 has excellent mechanical strength, extensibility and water–oxygen barrier properties and has broad application prospects in green packaging, lithium battery diaphragm and medical equipment materials. The correlation between the aggregation structure evolution and macroscopic comprehensive properties of copolymer PA56/512 under biaxial stretching has been demonstrated in this work. The structure of the random copolymerization sequence was characterized by ^13^C Nuclear magnetic resonance (NMR). The typical isodimorphism behavior of the co-crystallization system of PA56/512 and its BOPA-56/512 films was revealed by differential scanning calorimetry (DSC) and X-ray diffraction (XRD) tests. And the aggregation structure, including the hydrogen bond arrangement, crystal structure and crystal morphology of PA56/512 before and after biaxial stretching, was investigated by XRD, Fourier-transform infrared spectroscopy (FTIR) and polarized optical microscopy (POM) tests. Furthermore, the effect of the biaxially-oriented stretching process on the mechanical properties of PA56/512 has been demonstrated. In addition, a deep insight into the influence of the structure on the crystallization process and physical–mechanical performance has been presented. The lowest melting point at a 512 content of 60 mol% is regarded as a “eutectic” point of the isodimorphism system. Due to the high disorder of the structural units in the polymer chain, the transition degree of the folded chain (gauche conformation) is relatively lowest when it is straightened to form an extended chain (trans conformation) during biaxially-oriented stretching, and part of the folded chain can be retained. This explains why biaxially stretched PA56/512 has high strength, outstanding toughness and excellent barrier properties at the pseudo-eutectic point. In this study, using the unique multi-scale aggregation structure characteristics of a heterohomodymite polyamide at the pseudo-eutectic point, combined with the new material design scheme and the idea of biaxial-stretching processing, a new idea for customized design of high-performance multifunctional polyamide synthetic materials is provided.

## 1. Introduction

Polyamides (PA), known as nylon, containing an intermolecular hydrogen bond structure, have been widely used in engineering plastics, high-performance films and fibers due to their excellent wear and heat resistance, mechanical rigidity–toughness balance and processing performance [1,2]. PA6 and PA66 account for almost 90% of the global demand for polyamide applications, owing to the limited development of monomer solution and polymerization processing technology [3]. The monomer structure of 1,5-pentanediamine is similar to that of 1,6-hexanediamine, with a huge potential advantage of originating from natural raw materials such as straw and corn by biological fermentation [4,5,6]. Recently, Cathay Biotech Inc. has taken the lead in the mass production, preparation and commercialization of bio-based 1,5-pentenediamine, which promoted a new series of polyamides with a monomer composition of 5X to obtain new development opportunities and new market application prospects. In particular, the bio-based PA56, accompanied by better moisture permeability and flame retardation than the traditional commodities of PA66 and PA6, is more suitable for consumer application in the field of high-performance films and spun fibers [7,8] and is expected to realize large-scale petroleum-based replacement in the future. 

The major defect of poor mechanical toughness in PA56 restricts its application in the field of food packaging and lithium battery protection film, which has high impact and ductility requirements [9]. A physical blending modification has often been used as the simplest method to improve the performance of PA56, such as introducing a toughening agent with a flexible component like EPDM-g-MAH [10,11]. It is hard to effectively control the aggregation structure of the polymer components and the stability of each batch through the compounding process. Therefore, the most effective and controllable method is introducing long carbon chain polyamide components to improve the toughness of short-chain polyamides by means of copolymerization modification [12,13], which also has been widely used in co-polyester polymerization [14,15,16]. The common copolymerization types of polyamides include random, block and alternating copolymerization. Therein, random copolymerization is widely used as a result of its simple synthesis process and controllable reaction conditions. The completely random connection between the chemical units makes the phase behavior of polyamide more complex, involving the following types of crystallization: non-crystallization; only one kind of crystallization; independent crystallization; and co-crystallization [14,17,18]. As an ideal compatibility mode, the copolymerized components of the co-crystallization system have the ability to form a unified or similar crystal, which can be divided into isomorphism [19] and isodimorphism [20] behavior according to the difference in compatibility between each copolymerized unit. Many studies have shown that the regulation of specific properties of copolymers can be achieved by adjusting the ratio of the copolymer components, such as the combination properties of high strength and high toughness, high heat resistance and high toughness [12,21,22]. 

In addition, not only does the crystallization structure have a significant impact on the copolymer properties, but the processing method also plays a crucial role in the enhancement of copolymer properties. Casting stretching, blown film process and biaxially-oriented stretching methods are the common process solutions for film production. Particular focus has been on the biaxially-oriented film in recent years because of the advantages of fast production speed, large production capacity and high efficiency [23,24]. The crystalline ordering and crystallinity of the film obtained by bi-directional tensile orientation will also be significantly improved owing to the highly oriented orientation of the molecular chain, along with the improvement in tensile properties, barrier properties, dimensional stability, thickness uniformity and other properties of plastic film [25,26]. Biaxially-oriented polyamide (BOPA) [27], polypropylene (BOPP) [28] and polyester (BOPET) [29] have been used for flexible packaging applications and also new advanced applications such as the lithium battery.

We have systematically studied the structure, molecular weight, thermal properties and non-isothermal crystallization kinetics of PA56/512 resins with different monomer ratios in our previous studies [21]. Based on those, the preparation of biaxially-oriented PA56/512 films (BOPA-56/512) modified by long carbon chain copolymerization and the relationship between its microstructure evolution and macroscopic properties under biaxially-oriented conditions are demonstrated in this work. The long carbon chain copolymer PA56/512 was prepared by melt polymerization based on 1,5-pentanediamine, adipic acid and dodecanedioic acid. The BOPA-56/512 was stretched by its extruded casting film. The structure of the random copolymerization sequence was characterized by ^13^C NMR. The thermal properties, crystal structure, hydrogen bond arrangement and crystal morphology of PA56/512 before and after biaxial stretching were characterized by DSC, XRD, FTIR and POM tests. Furthermore, the effect of bidirectional stretching on the mechanical properties of PA56/512 was studied using different physical–mechanical measurements. This work is expected to provide theoretical guidance for the development of bio-based long carbon chain copolymerized biaxially-oriented polyamide with excellent comprehensive performance. 

## 2. Experimental Section

### 2.1. Materials 

1,5-pentanediamine was purchased from Cathay Biotech Inc. (Shanghai, China). Adipic acid was purchased from Sinopec Yangzi Petrochemical Co., Ltd. (Nanjing, Jiangsu, China). Dodecanedioic acid and trifluoroacetic acid-d (isotopic) were purchased from Aladdin Industrial Corporation (Shanghai, China). All reagents used were of analytical purity. All solutions were prepared by using deionized water. 

### 2.2. Preparation of Copolymer PA56/512 

**Scheme 1:**
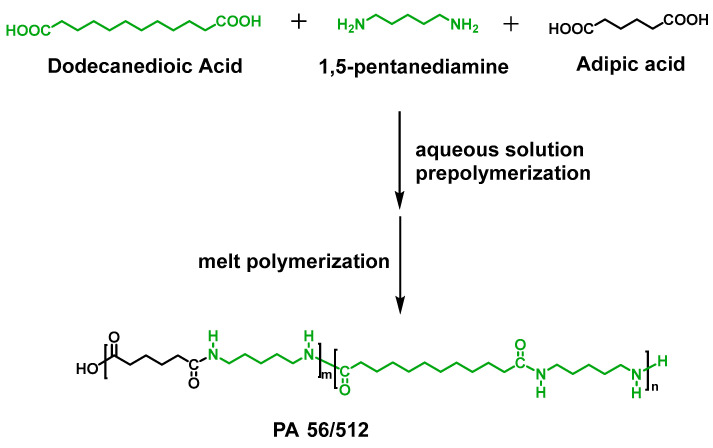
Synthetic route of PA56/512.

Scheme 1 presents the synthetic route of PA56/512. The copolymer PA56/512 prepared by monomeric adipic acid and dodecanedioic acid with molar ratios of 8:2, 7:3, 6:4, 5:5, 4:6, 3:7, 2:8 was named as samples #2, #3, #4, #5, #6, #7, #8, respectively. Pure PA56 and pure PA512 were named samples #1 and #9, respectively. The process of preparing PA56/512 resin by neutralization and polymerization reaction is consistent with our previous study [21]. 

Neutralization Reaction. The adipic acid and dodecanedioic acid, with a total weight of 2.5 kg, were prepared in a 50% mixed aqueous solution according to the set molar ratio (8:2, 7:3, 6:4, 5:5, 4:6, 3:7, 2:8). Then, 1,5-pentanediamine was added dropwise to the above mixed aqueous solution under vigorous stirring at 80 °C. A transparent aqueous solution was obtained when the pH of the whole solution was regulated at 7.8.

Polymerization reaction. The above solution was transferred to a 10 L autoclave, and the air in the autoclave was replaced with nitrogen 3–5 times. The reactor was heated at 155 °C to drain 1.1 kg of water; the temperature was increased to 200 °C, and the pressure was maintained at 1.5 MPa for an hour. After the autoclave was heated to 260 °C with 1.5 MPa pressure, the vapor in the autoclave was gradually released to reduce the pressure to normal. The system was vacuumed and reacted at −0.08 MPa for 1 h. Finally, the PA56/512 resin was obtained after the discharging, cooling and granulating.

### 2.3. Preparation of BOPA-56/512 

**Figure 1:**
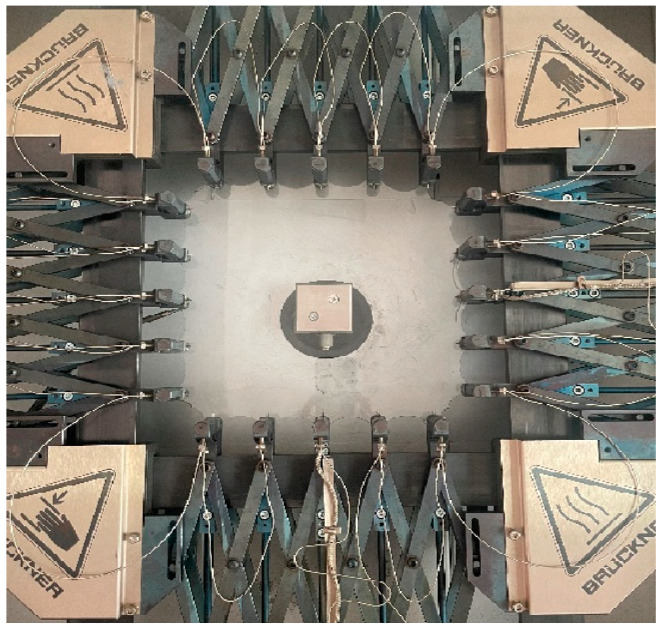
Diagram of the biaxially-oriented stretching process of BOPA-56/512.

First, the copolymerized PA56/512 films were prepared by an extrusion–casting mechanism (FDHU-35, Guangzhou Putong Instrument, Guangzhou, China). The extrusion temperature was 20 °C higher than the melting point of the polymer, and the extrusion speed was 20 r/min. After extrusion, the films were cooled on the casting roll at 30 °C. Then, the cast films were cut into square slices of 100 × 100 mm and biaxially stretched with a biaxial tensile testing machine (KARO^®^ 5.0, Bruckner Group GmbH, Siegsdorf, Germany). And the stretching time, stretching ratio, stretching rate, preheating time, preheating temperature, heat setting temperature and setting time were set to 0.5 s, 2 × 2, 100%, 90 s, the crystallization peak temperature below 10 °C, the incipient melting temperature below 20 °C, 60 s, respectively. The original samples #1~#9 were processed into the corresponding biaxially-oriented polyamide films (BOPA-56/512) and named #1′~#9′, respectively. The diagram of the stretching process of BOPA-56/512 is shown in Figure 1. 

The characterization methods and details of PA56/512 and BOPA-56/512 are shown in the Supplementary Materials. 

## 3. Results and Discussion

### 3.1. Chemical Structural Characterization of PA56/512 

**Figure 2:**
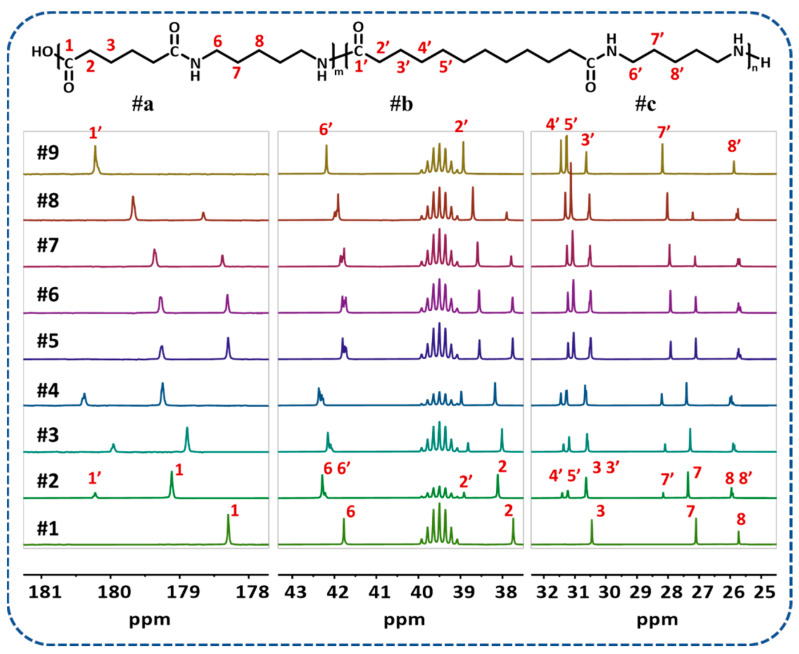
^13^C NMR spectra of PA56/512 with different composition ratios.

The ^13^C NMR spectra of PA56/512 with different composition ratios are shown in Figure 2. Region #a (181–178 ppm) represents the characteristic peak positions of the C (1, 1′) atom of diacids in PA56/512. All α-C of diamines (6, 6′) and diacids (2, 2′) in PA56/512 are displayed in region #b (43–37 ppm) and the β/γ-C of diamines (7, 7′, 8, 8′) and β/γ/δ-C of diacids (3, 3′, 4, 4′, 5, 5′) appear in region #c (32–25 ppm). Furthermore, the characteristic peak intensity of C atoms in diacids (1), α-C in diacids (2) and β-C in diamines (7) in 56 units of PA56/512 decreased gradually with an increase in 512 content. On the other hand, the peak intensity of the corresponding C atom position in the 512 unit (1′, 2′, 7′) of PA56/512 increased gradually, which is consistent with the feeding ratio, demonstrating the successful preparation of a PA56/512 random copolymer. 

### 3.2. Effect of Bidirectional Stretching on Thermal Properties of PA56/512 

**Figure 3:**
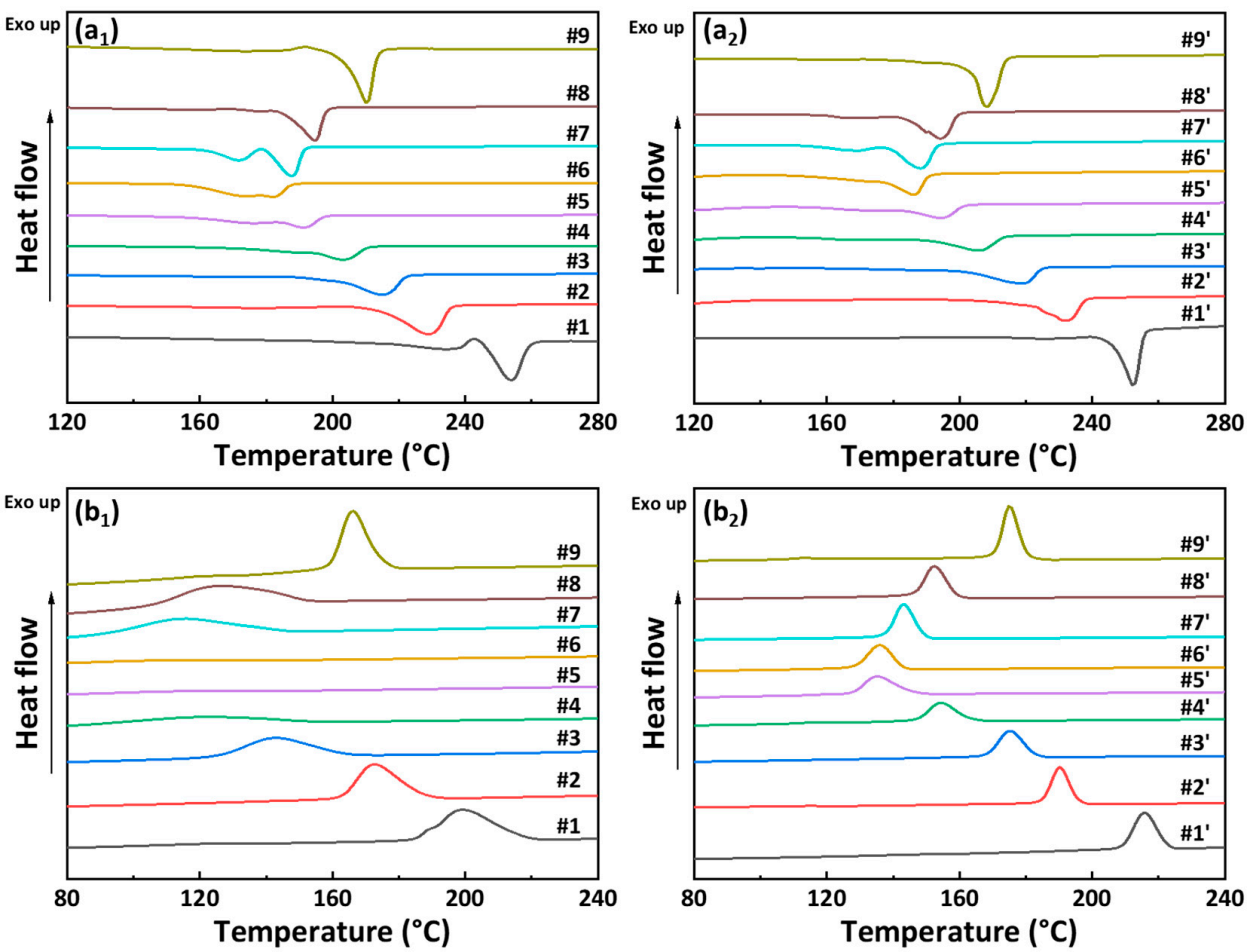
(**a_1_**) [21] The second heating DSC curves of PA56/512 resin with different composition ratios, (**a_2_**) the first heating DSC curves of BOPA-56/512 with different composition ratios, the crystallization curves of (**b_1_**) [21] PA56/512 resin, (**b_2_**) BOPA-56/512 with different composition ratios. All the samples are set at heating/cooling rates of 10 °C/min.

**Figure 4:**
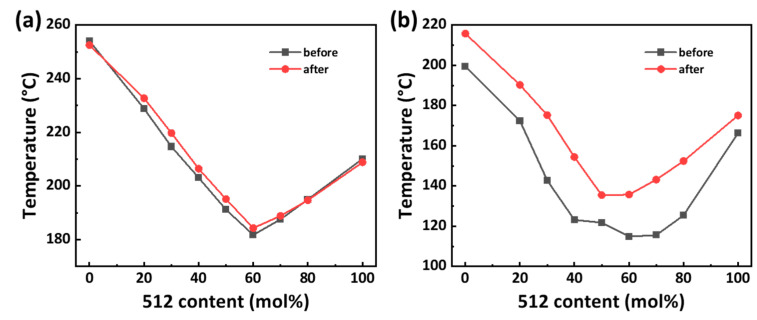
(**a**) [21] The melting temperature curves and (**b**) crystallization temperature curves of PA56/512 before and after biaxial stretching with different content of 512.

The melting and crystallization curves of PA56/512 before and after biaxial stretching with different composition ratios from the DSC tests are shown in Figure 3. It can be clearly seen from Figure 3a_1_,a_2_ that the melting points of both decrease first and then increase with an increase in 512 content, according to the typical isodimorphism behavior [30]. And the crystallization curves display a similar trend. Figure 4 depicts the melting and crystallization temperature curves of PA56/512 before and after biaxial stretching with different contents of 512. The lowest melting point with a 512 content of 60 mol% is regarded as a “eutectic” point of the isodimorphism system [31], which is considered to be the dividing point of the dominant crystal form of the 56 or 512 homopolymers. Furthermore, it can be observed that the melting point of PA56/512 hardly changes before and after biaxial stretching, which means that the biaxial stretching behavior will not alter the isodimorphism properties of the copolymerization system of PA56/512. 

Compared with Figure 3b_1_,b_2_, BOPA-56/512 shows an obvious exothermic crystal peak around the “eutectic” point along with a relatively narrow crystallization peak shape. Meanwhile, PA56/512 without biaxial stretching are vice versa. Otherwise, the crystallization peak position of BOPA-56/512 moves to the higher temperature position by more than 10 °C compared to PA56/512 without biaxial stretching. It can be concluded that the crystallization capacity of PA56/512 is obviously improved by bidirectional stretching. 

The relevant studies on the crystal transition of polyamides indicate that the hydrogen bond of polyamides will not completely be destroyed in the melting process, and the retention degree of the hydrogen bond structure is related to the degree of crystallization perfection [32,33,34]. Studies on the melt-memory effect demonstrate that the residual hydrogen bond structure or other ordered molecular chain structure in polyamide melt can be used as the initial nucleation point and play a role in inducing crystallization during melt–cooling crystallization [35,36]. Therefore, it can be inferred that the hydrogen bond strength and molecular chain arrangement orderliness of PA56/512 are effectively improved during the bidirectional tensile process, which means the trans-conformation molecular chain with high orderliness is more likely to be retained during the melting process, so that the system of BOPA-56/512 has a higher crystallization temperature and stronger crystallization capacity during the cooling crystallization process. 

### 3.3. Effect of Biaxial Stretching on Mechanical and Barrier Properties of PA56/512 

**Figure 5:**
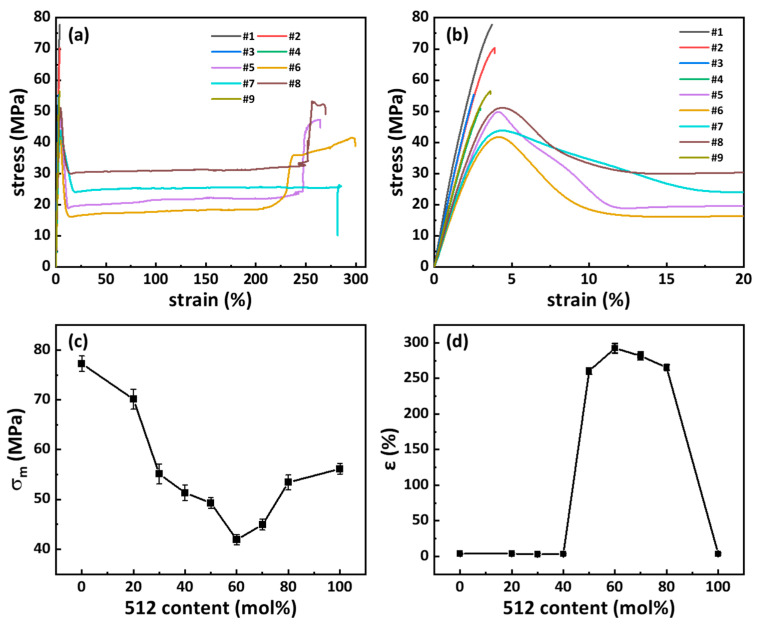
The mechanical properties curve of PA56/512 resin: (**a**) tensile stress–strain curve, (**b**) local enlarged tensile stress–strain curve, (**c**) tensile strength with different contents of 512, (**d**) tensile elongation yield with different contents of 512.

The mechanical properties curve of PA56/512 resin without biaxial stretching is shown in Figure 5 and Figure S1. The curve trends of tensile strength, bending strength and bending modulus of PA56/512 with different content of 512 are similar to those of its melting point, which decreased first and then increased, reaching the lowest value at the “eutectic” point with a 512 content of 60 mol%. Meanwhile, the mechanical toughness indexes, such as tensile elongation yield and notched impact strength of the cantilever beam, displayed the opposite trend. PA56/512 had the highest mechanical toughness and the lowest mechanical strength at the “eutectic” point, with a tensile elongation yield of 293% and a tensile strength of 42 MPa, which is common in the random copolyester system, and also related to the “compatibility” and “competition” of random copolymerization units [14,30]. The crystallization of the 56 and 512 structural units at the “eutectic” point presents the most obvious inhibition effect on each other, resulting in the loosest interface between the crystalline zone and the amorphous zone and the best energy absorption and deformation ability under external force, leading to the highest tensile elongation yield and notched impact strength of PA56/512 at this point. 

**Figure 6:**
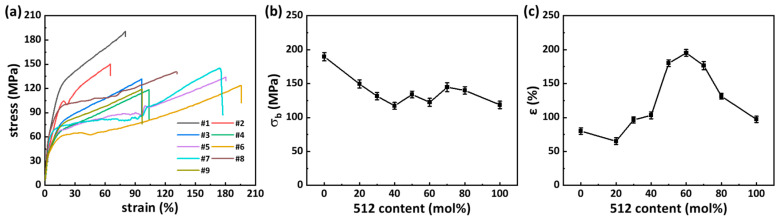
The tensile properties of BOPA-56/512 films: (**a**) tensile stress–strain curve, (**b**) breaking strength with different contents of 512, (**c**) tensile elongation yield with different contents of 512.

Figure 6 shows the tensile properties of BOPA-56/512 films. It can obviously be seen in Figure 6b that the breaking strength of BOPA-56/512 was several times higher than that of PA56/512 without bidirectional stretching, attributing to the enhancement of hydrogen bond strength and conformational order of BOPA-56/512 by bidirectional stretching. The variation trend of tensile elongation yield with different 512 contents of BOPA-56/512 was the same as that of the PA56/512 without bidirectional stretching, which also reflects that the bidirectional stretching does not change the characteristic of isodimorphism in these co-crystallization systems. The tensile elongation yield of homopolymer PA56 and PA512 was significantly higher than that of PA56/512 without bidirectional stretching, which may be related to the large decrease in spherulite size. 

**Figure 7:**
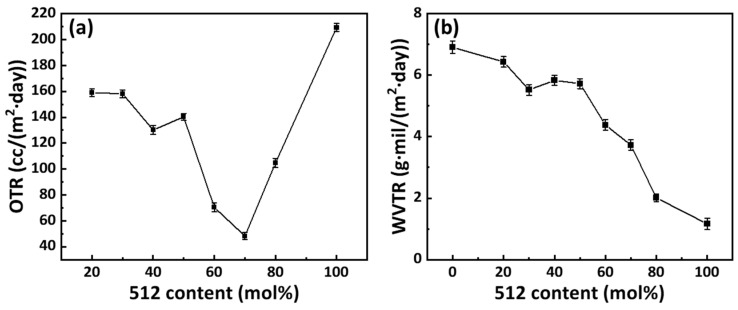
Barrier properties of BOPA-56/512 films: (**a**) oxygen permeability curve; (**b**) water vapor permeability curve.

**Figure 8:**
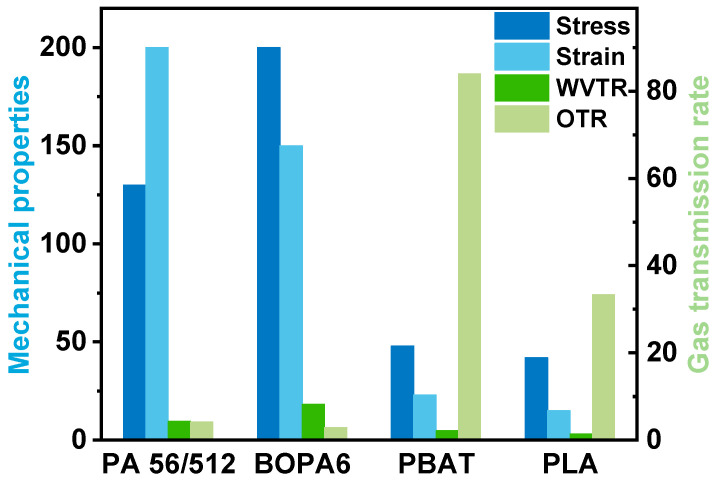
Comparison of comprehensive properties of various barrier materials.

The barrier properties of PA56/512 with different composition ratios after biaxial stretching are shown in Figure 7. As can be seen, with an increase in the molar ratio of 512, its barrier ability to water vapor continuously improved, while its barrier capacity to oxygen first increased, followed by a decrease, and it had the best barrier performance to oxygen near the “eutectic” point. It has been shown that the water vapor barrier properties of polyamide materials are closely related to the density of hydrogen bond [37]. The lower the density of the hydrogen bond, the higher the water vapor barrier property. The barrier properties of oxygen are closely related to the crystalline aggregate structure of the material. When lamellar graphene or montmorillonite with a large specific surface area is added to polyamide material, its oxygen barrier performance will be greatly improved [27]. At the same time, the crystal aggregation structure of the biaxial stretching melting polyamide will also undergo spherulite fragmentation and re-formation of stretching extended chain crystal microstructure evolution process [38], and its oxygen barrier property will be significantly improved. A comparison of the comprehensive properties of various barrier materials (Figure 8) shows that the BOPA-56/512 prepared in this study had excellent barrier properties, mechanical strength at pseudo-eutectic point, and its barrier properties to oxygen and mechanical strength were comparable to that of commercial BOPA6, and it had better mechanical toughness and barrier properties to water vapor. In addition, the comprehensive performance of PA56/512 was significantly superior to that of PLA and PBAT bio-based synthetic materials, and it can be used as a new generation of green and high-performance barrier packaging materials. In order to clarify the high-performance characteristics of biaxially-oriented PA56/512, the multi-scale aggregation structure of PA56/512 was further studied.

### 3.4. Effect of Biaxial Stretching on Crystal Structure of PA56/512 

**Figure 9:**
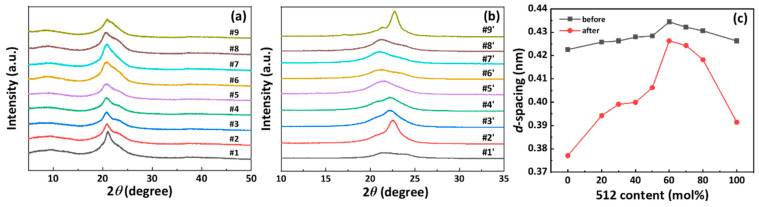
XRD spectra of PA56/512 (**a**) before and (**b**) after biaxial stretching with different composition ratios, (**c**) the d-spacing curves of PA56/512 before and after biaxial stretching with different contents of 512.

The XRD pattern of PA56/512 before and after biaxial stretching is shown in Figure 9a,b for crystal analysis of the system. All the samples presented a single peak at a similar diffraction angle (2*θ*) of 20.5°~22.5°, corresponding to the (010) planes of PA56/512, whose crystal pattern belongs to the typical pseudohexagonal γ form [39,40]. The corresponding value of d-spacing d_010_ representing the distance between the hydrogen bond planes was derived by the Bragg equation, and the d-spacing curves of PA56/512 before and after biaxial stretching with different contents of 512 are drawn in Figure 9c. It can be observed that the d_010_ increased first and then decreased with an increase in the 512 content, reaching a maximum value at the “eutectic” point with a 512 content of 60 mol%. According to the crystallography principle, the crystallization of polymers follows the densest packing principle [41,42]. The higher the crystal order degree, the higher the crystal packing density and the shorter the hydrogen bond plane spacing [43]. The high molecular symmetry of PA56 and PA512 homopolymers results in a highly ordered crystalline structure of themselves, and thus, the #1 and #9 samples have relatively low hydrogen bond plane spacing. In other words, the order degree of the crystals formed by random copolymerization of PA56 and PA512 corresponding to the #2~#8 samples was lower than that of their own original homopolymer. When 56 was dominant in the copolymer system, the insertion of 512 resulted in a lower orderliness than the original PA56 system. Similarly, when 512 crystals dominated, the insertion of 56 caused the copolymer system to be less ordered than the original PA512 system. Therefore, it can be explained that the hydrogen bond plane spacing of PA56/512 increased first and then decreased with an increase in 512 contents, and the whole system reached the maximum hydrogen bond plane spacing and the lowest crystal orderliness at the “eutectic” point with a 512 content of 60 mol%. 

Compared the two d-spacing curves of PA56/512 before and after biaxial stretching with different contents of 512 in Figure 9c, it can be seen that the d_010_ spacing of PA56/512 after biaxial stretching was significantly smaller than that before biaxial stretching, resulting from the improvement in crystal order degree and packing density based on the bidirectional tensile force through conformational transformation. 

In addition, the crystalline structure of polyamide is directly related to the intermolecular hydrogen bond. The changes in hydrogen bond strength and molecular chain conformation of PA56/512 before and after biaxial stretching were studied by FTIR analysis. Figure S2 depicts the comparison of FTIR spectra of PA56/512 before and after biaxial stretching with different composition ratios. The similar peaks presented in sample #1 and #1′ (#2 and #2′, #3 and #3′, etc.) and the presence of typical Amide I-V peaks indicate that all PA56/512 (sample #1-#9, #1′-#9′) prepared had typical polyamide structure [44]. The characteristic peak observed around 3300 cm^−1^ belonged to the stretching vibrations of N−H bonds. The position of the above characteristic peak of PA56/512 after bidirectional stretching was shifted to the lower wavenumber. Relevant studies have confirmed that the higher the hydrogen bond strength, the more limited the conformation movement of molecular chains, and the lower the wavenumber of related characteristic peaks [45,46,47]. Therefore, the fact that the hydrogen bond strength of PA56/512 was enhanced and the conformation movement of the molecular chain was limited under the action of the biaxial tensile force field is consistent with the DSC analysis results. 

**Figure 10:**
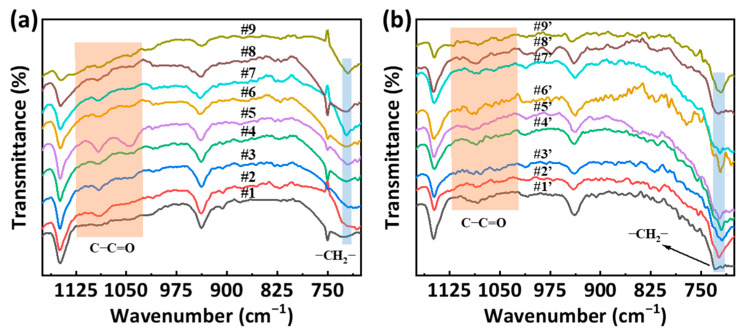
FTIR spectra in the fingerprint region of PA56/512 (**a**) before and (**b**) after biaxial stretching with different composition ratios.

The FTIR spectra in the fingerprint region of PA56/512 before and after biaxial stretching with different composition ratios are shown in Figure 10. The characteristic peaks at 940 cm^−1^ and 1120~1020 cm^−1^ represent the C−C=O stretching vibration in the crystal region and amorphous region of PA56/512 molecular chain, respectively [33]. It can be observed that the peak position (940 cm^−1^) of the C−C=O stretching vibration in the crystal region was almost unchanged before and after biaxial stretching. The peak strength of C−C=O stretching vibration in the amorphous region (1120~1020 cm^−1^) of PA56/512 after biaxial stretching was relatively not obvious and weak, along with the trend of migration to the lower wavenumber. Based on the above analysis, it can be inferred that the crystallization capacity and the ordering of molecular chains in the amorphous region of PA56/512 will be significantly improved after biaxial stretching, corresponding to the conclusion of DSC tests. Furthermore, the closer to the “eutectic” point, the higher the peak intensity of C−C=O stretching vibration of PA56/512 in the amorphous region, further illustrating the worst crystallization ability and the lowest crystal orderliness at the “eutectic” point. 

The rocking vibration of methylene with trans conformation can be seen at a wavenumber of 720 cm^−1^. The characteristic peak of methylene rocking vibration for PA56/512 without biaxial stretching was obvious only in the case of a high 512 molar content. However, the methylene rocking vibration peak of PA56/512 after biaxial stretching appeared in any copolymerization ratio. It can be explained by the following related studies on the crystallization properties of odd-even polyamides. 

Sanjay et al. [48] found that the longer the saturated carbon chain, the stronger the molecular chain motion and the faster the conformational conversion rate of the semi-aromatic polyamide so that the rocking vibration peak of methylene in PA56/512 appears obviously under a high 512 molar content. Puiggali et al. [49] found that all amide groups can participate in the formation of 100% hydrogen bonds after a slight rotation from the amide plane of PA56 and the plane defined by methylene carbon atoms. The unique twisted hydrogen bond structure of the odd–even polyamides is completely distinct from the all-transplanar hydrogen bond structure of the even–even polyamides, which requires relatively high energy or a dilute solution for a long time to form a perfect high-orderliness monoclinic α crystal form [50], while it can only form a pseudo-hexagonal γ crystal form with a low ordering degree in the general melt–cooling process [51]. Therefore, it can be inferred that copolymerized PA56/512 with any copolymerization ratio after biaxial stretching can absorb more external energy to order its molecular chain conformation and form more trans conformations. 

### 3.5. Effect of Biaxial Stretching on Crystal Morphology of PA56/512 

Figure S3 displays the POM images of PA56/512 with different composition ratios before biaxial stretching. It can be seen that the spherulite size of PA56/512 showed a process of rapidly decreasing and then increasing with an increase in 512 contents in the copolymer. And the spherulite structure was almost invisible at the ratio of the “eutectic” point, which can be explained by the “compatibility” and “competition” between the copolymerized structural units of random copolymers [16]. In other words, the two copolymeric structural units in the random copolymer are arranged randomly, and the crystallization of their copolymeric structural units is constrained by each other so that the random copolymer PA56/512 is difficult to grow into a large-size spherulite structure through effective molecular chain folding. 

**Figure 11:**
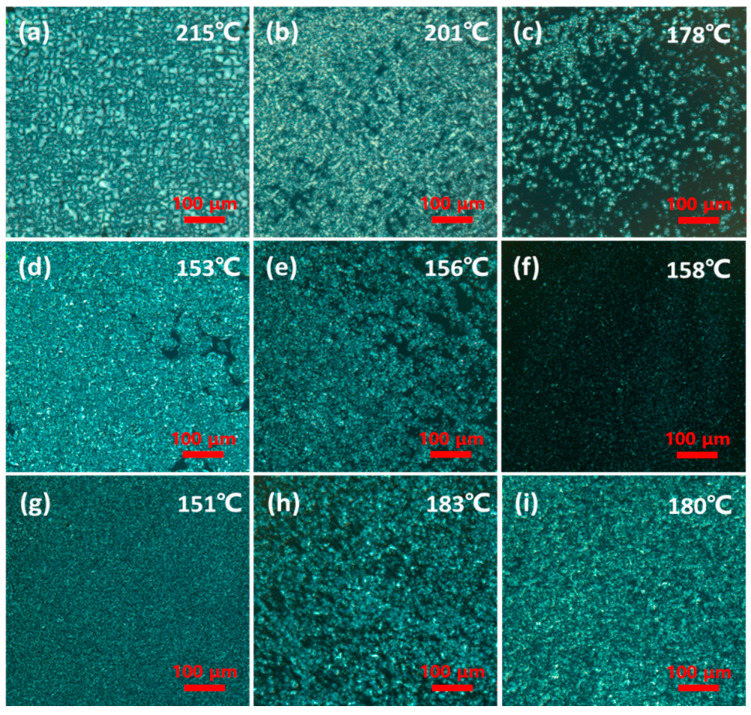
POM images of PA56/512 after biaxial stretching: (**a**) #1′, (**b**) #2′, (**c**) #3′, (**d**) #4′, (**e**) #5′, (**f**) #6′, (**g**) #7′, (**h**) #8′, (**i**) #9′.

Figure 11 shows the POM images of PA56/512 with different composition ratios after biaxial stretching. The variation trend of spherulite size and copolymerization composition of PA56/512 after bidirectional stretching was the same as that without biaxial stretching, while the spherulite size increased significantly around the “eutectic” point. It can be attributed to the more ordered molecular chain conformation and stronger hydrogen bond strength after biaxial stretching. Even in the melting process, these ordered structures will be retained due to the melt-memory effect [35,36]. Therefore, it is conducive to the formation of more perfect spherulite in the process of melting–cooling. The spherulite size of homopolymer PA56 and PA512 decreased with the increased nucleation density, attributing to the enhancement of the melt-memory effect after bidirectional stretching. 

### 3.6. Molecular Mechanism Explanation of PA56/512 with High Strength, Toughness and Barrier Properties

**Figure 12:**
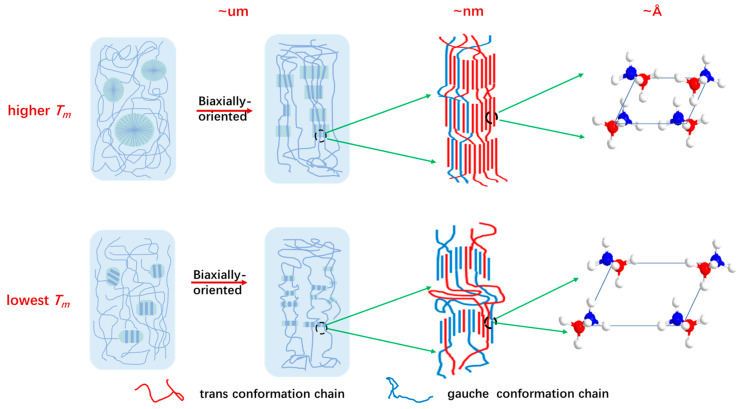
Molecular mechanism explanation of high-performance PA56/512.

Biaxially-stretched PA56/512 ha high strength, high toughness and excellent water-oxygen barrier properties at the “eutectic” point, which can be explained by the multi-scale aggregation structure evolution of random copolymers under the tensile field (Figure 12). As previous studies have shown, the random copolymer PA56/512 is a typical isomorphism co-crystallization system. The closer the copolymerization composition is to the “eutectic” point, the lower the order of the polymer chain and the lower the crystallization ability (Figure 3). It can be considered that when the copolymerization composition is closer to the “eutectic” point, the amorphous region is larger, while the crystallization region is only scattered in the continuous amorphous region [14]. This can be verified by the study of crystal morphology (Figure S3 and Figure 11) in this paper.

According to existing studies on stretch-induced crystallization [38,52,53], polymer chains such as polyolefin or random copolyester are prone to stress-induced conformation motion, and more extended chains (trans conformation) are formed from the initial folded chain (gauche conformation). At the same time, microstructure evolution such as slipping-fragment-recrystallization will occur in the crystallization zone, from the initial large spherulite to fine irregular crystal morphology [38]. The conformational transformation (Figure S2 and Figure 10) and the crystal morphology changes (Figure S3 and Figure 11) of the random copolymer PA56/512 under the static- and biaxial tensile force field in this study also verify it, which is also the fundamental reason for the strength increase in PA56/512 after biaxial tensile. Furthermore, due to the lowest order of polymer chains and the most amorphous regions at the “eutectic” point [15,16], it can retain as many folded chains (gauche conformation) as possible under the biaxial-tensile force field, so there are relatively more possibilities for molecular sliding movement during the tensile or impact process of materials. In addition, the size of the crystallization zone induced by stretching is the smallest and most irregular, which makes the path of oxygen in this aggregated structure more complicated. This is the most important reason why PA56/512 melted at the “eutectic” point can still maintain excellent mechanical toughness (elongation at break) and the highest barrier property after biaxial stretching.

## 4. Conclusions

The bio-based copolymer PA56/512 with different composition ratios was synthesized by one-step melt polymerization with monomers of 1,5-pentanediamine, adipic acid and dodecanedioic. The BOPA-56/512 was prepared using the extrusion casting process and biaxially-oriented processing in the lab. The random copolymer structure of PA56/512 was verified by a ^13^C NMR spectrum. The melting and the crystallization curves of PA56/512 and BOPA-56/512 with different composition ratios found in DSC and XRD tests revealed their typical isodimorphism behavior, which obtained the lowest melting point at the “eutectic” point with a 512 content of 60 mol%. Moreover, the biaxial tensile force field can significantly improve the crystallization capacity of PA56/512 and form a more compact crystalline stacking structure, manifested in the obvious reduction in d-spacing d_010_. Mechanical and mechanical properties studies showed that PA56/512 at the “eutectic” point has excellent water–oxygen barrier properties, high tensile elongation at break, and high mechanical strength properties after biaxially-oriented stretching and its comprehensive properties are fully comparable to existing commercial BOPA6 materials, and has a very large application potential.

FTIR and POM tests showed that biaxial stretching PA56/512 will form more trans conformations through conformational movement, which will further straighten the polymer chain so that its hydrogen bond strength will be strengthened, and a smaller and irregular crystal shape will be formed, which is completely different from the static large-size spherulites. At the same time, PA56/512 at the “eutectic” point can retain relatively more folded chains (gauche conformation) during the biaxial tensile process due to the high disorder of the molecular chain structure units, so it has the best comprehensive properties. This also explains the high strength, high toughness and excellent barrier properties of biaxially stretched PA56/512 at the molecular level. In this study, using the isomorphism co-crystallization structure characteristics of polyamide random copolymer, combined with the new material design scheme of the biaxial stretching processing method, a new idea has been provided for the customized design of high-performance multifunctional polyamide synthetic materials.

## Data Availability

Data are contained within the article.

## Supplementary Materials

The following supporting information can be downloaded at: https://www.mdpi.com/article/10.3390/polym16081176/s1, Figure S1: The mechanical properties curve of PA56/512 resin: (a) bending strength with different content of 512, (b) bending modulus with different content of 512, (c) notched impact performance of cantilever beam with different content of 512 in 23 °C, (d) notched impact performance of cantilever beam with different content of 512 in −30 °C. Figure S2: FTIR spectra of PA56/512 before and after biaxial stretching with different composition ratios: (a) #1 and #1′, (b) #2 and #2′, (c) #3 and #3′, (d) #4 and #4′, (e) #5 and #5′, (f) #6 and #6′, (g) #7 and #7′, (h) #8 and #8′, (i) #9 and #9′. Figure S3: POM images of PA56/512 before biaxial stretching: (a) #1, (b) #2, (c) #3, (d) #4, (e) #5, (f) #6, (g) #7, (h) #8, (i) #9.
