# Supplementary material for: Pseudo-Eutectic of Isodimorphism to Design Biaxially-Oriented Bio-Based PA56/512 with High Strength, Toughness and Barrier Performances
Source: Polymers (Basel). 2024 Apr 22;16(8):1176. doi: 10.3390/polym16081176 (PMC11053481; doi:10.3390/polym16081176)
Supplement: Supplementary file 1 [file polymers-16-01176-s001.zip › polymers-2927454-supplementary.pdf]

Supporting information

Pseudo-eutectic of isodimorphism to design biaxial oriented bio-based PA56/512 with high strength, toughness and barrier performances

Diansong Gan[a,b], Yuejun Liu[a,1], Tianhui Hu[b], Shuhong Fan[a], Lingna Cui[a], Guangkai Liao[a], Zhenyan Xie[a], Xiaoyu Zhu[a] Kejian Yang[c,1].

[a] Key Laboratory of Advanced Packaging Materials and Technology of Hunan Province, School of Packaging and Materials Engineering, Hunan University of Technology, Zhuzhou 412007, China

[b] Zhuzhou Times Engineering Plastics Industrial Co., LTD. Hunan Zhuzhou, 412008, China

[c] School of Chemical Engineering and Light Industry, Guangdong University of Technology, Guangzhou510006, P. R. China

Corresponding author.
yjliu_2005@126.com (Y. J. Liu)
yangkj6@mail2.sysu.edu.cn (K. J. Yang)

Contents:
Total number of pages: 7
Total number of Figures:3
Total number of Reference: 8

Characterization

^{13}C NMR was measured by a Bruker AVIII 500HD (500 MHz) instrument with the solvent of dimethyl sulfoxide-d_6 and hexafluoroisopropanol to analyze the chemical structure of PA56/512.

The thermal performance of all samples was measured by differential scanning calorimetry (DSC 214, NETZSCH). All samples were heated from 25°C to 280°C with a heating rate of 10°C/min. After holding at 280°C for 3 min, the program was set to cool down to 25 °C at the set cooling rate of 10°C/min. After holding at 25°C for 3 min, the second heating program was the same as the first time. All the DSC tests above were of nitrogen atmosphere.

The oxygen permeability test was carried out on permeabil-ity tester (Ox-Tran@Model 2/21, USA Mocon Company) at 23℃and 50% relative humidity and 1 atm pressure according to ASTM D 3985. A 98% nitrogen (N_2) and 2% hydrogen (H_2) mixture was used as the carrier gas and 100% oxygen (O_2) was used as the test gas. The test was ended when the oxygen flux changed by <1% during a 40 min test cycle. The reported data have been normalized by the films' thickness and are an average of four tests. The water vapour transmission rate (WTR) of PA56/512 was studied by Labthink W3-060 water permeability tester at certain relative humidity S5 (RH=90%) and temperature (38°C). The distilled water was used. The testing range was 0.1-10000 $g/m^2 \cdot 24h$. The water permeability rate was calculated by measuring the weight of water which passed the films. The testing films were prepared following the same method as gas barrier testing with the diameter of 74 mm and the permeability surface area of 33 cm^2 .

X-ray diffractometer (XRD, D8 ADVANCE, BRUKER) and Fourier transform infrared spectrometer (FTIR, PerkinElmer Frontier) were carried out to obtain the crystal structure and hydrogen bond arrangement information of copolymer PA56/512 and its BOPA.

Polarized Optial Microscope (POM, Leica DM4P, Germany) was used to observe

the crystal morphology of copolymer PA56/512 and its BOPA. All samples were heated to 30°C above the melting point with a heating rate of 5°C/min and stayed for 3 min. Then the growth of spherulite was observed at a cooling rate of 5°C/min.

The electronic universal testing machine (BTC-EXMUL TI-PAC2, Zwick/Roell, Germany) was used to test the tensile and bending properties of the samples. According to the standard of GB/T 1040-2006, the 1A dumbbell samples were measured at an ambient temperature of 23°C with the tensile rate of 20 mm/min. Based on the standard of GB/T 9341-2008, the samples size of 80 mm × 10 mm × 4 mm were measured at an ambient temperature of 23°C with a span of 64 mm and a rate of 2 mm/min. And the impact properties of the samples were tested by a cantilever beam impact tester (TS-PIT501JA, Shenzhen Wance test equipment, Shenzhen, China) at ambient temperatures of 23°C and −30°C according to the standard of GB/T 1843-2008. The above mechanical properties were tested five times for each sample, and the average value was finally taken.

The preparation process of PA56/512 resin injection molding splines is as follows. The fully dried samples #1 ~ #9 were moulded into splines by injection molding machine (ES300 DUO, ENGEL). The injection molding temperature is set according to the respective melting point of the resin. The injection temperature of #1 is set to 245°C, 260°C, 280°C, 280°C and 275°C. The injection temperature of #2 is set to 220°C, 235°C, 255°C, 255°C and 245°C. The injection temperature of #3 is set to 205°C, 220°C, 240°C, 240°C and 235°C. The injection temperature of #4 is set to 195°C, 210°C, 230°C, 230°C and 225°C. The injection temperature of #5 is set to 180°C, 195°C, 215°C, 215°C and 210°C. The injection temperature of #6 is set to 170°C, 185°C, 205°C, 205°C and 200°C. The injection temperature of #7 is set to 175°C, 190°C, 215°C, 215°C and 205°C. The injection temperature of #8 is set to 185°C, 200°C, 220°C, 220°C and 215°C. The injection temperature of #9 is set to 200°C, 215°C, 235°C, 235°C and 230°C. The screw speed is set to 50 r/min, the injection back pressure is 80 MPa, and the pressure holding time is 10 s.

Mechanical properties of PA56/512 copolymer

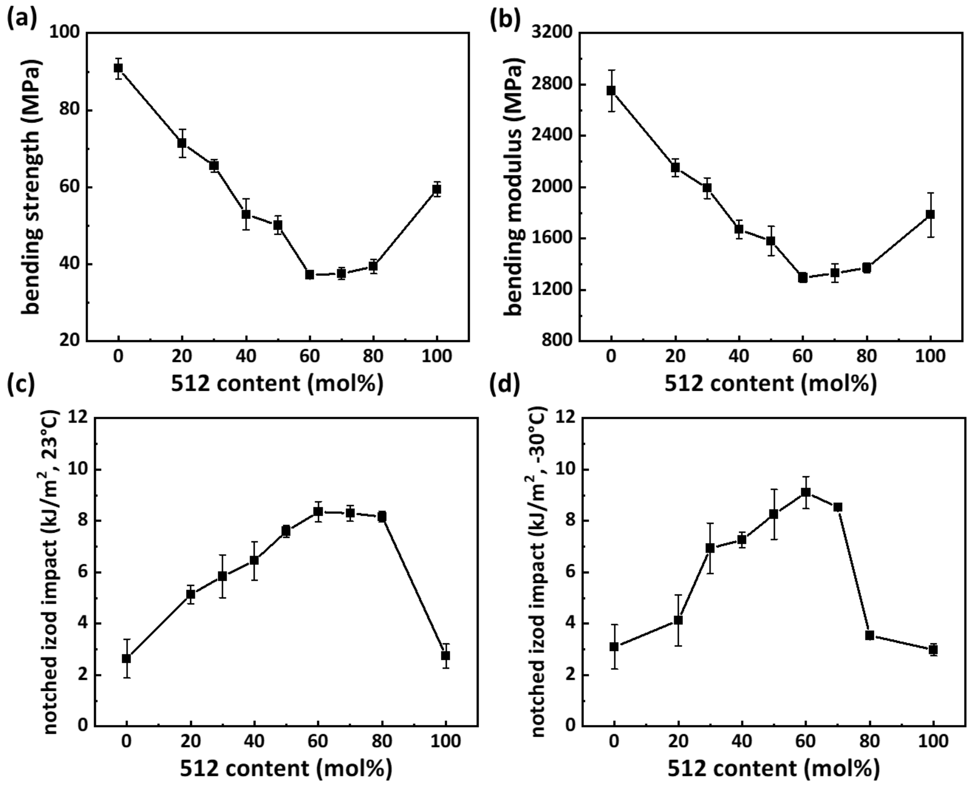

Fig. S1 The mechanical properties curve of PA56/512 resin: (a) bending strength with different content of 512, (b) bending modulus with different content of 512, (c) notched impact performance of cantilever beam with different content of 512 in 23°C, (d) notched impact performance of cantilever beam with different content of 512 in −30°C

FTIR spectra of PA56/512 copolymer before and after biaxial stretching

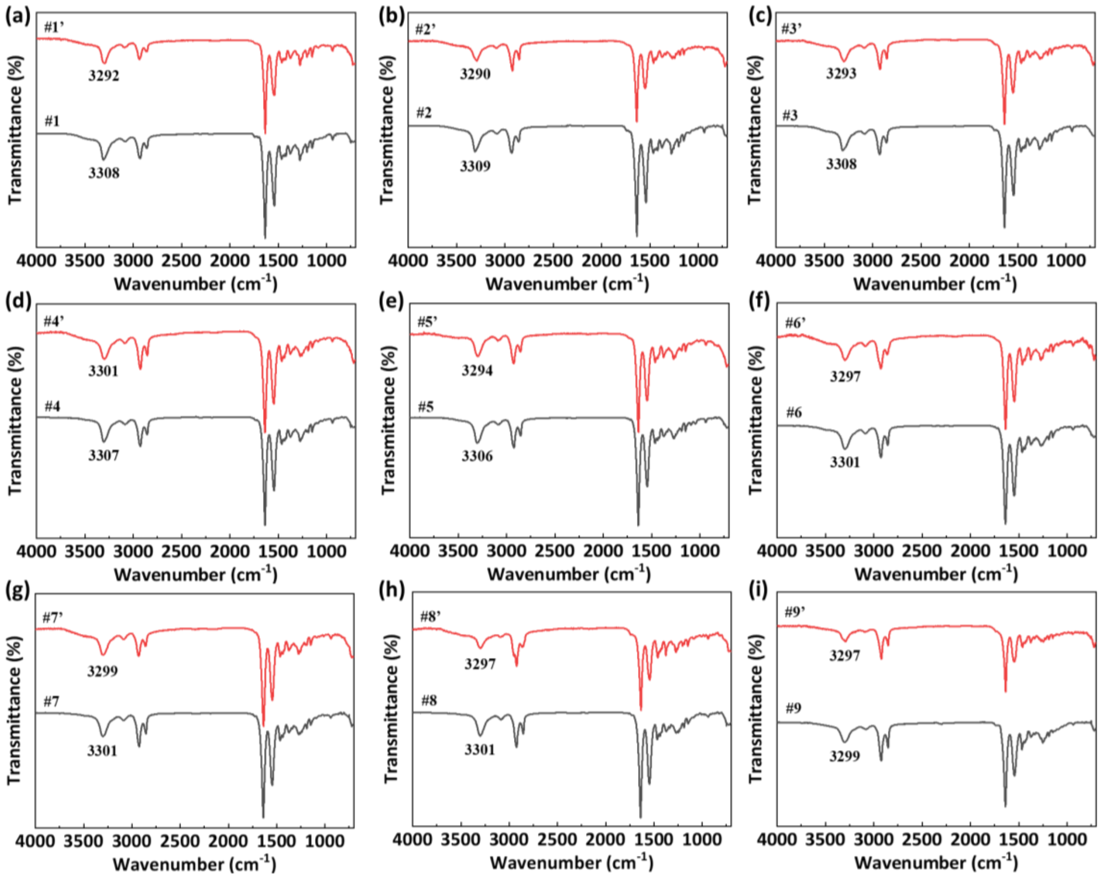

Fig. S2 FTIR spectra of PA56/512 before and after biaxial stretching with different composition ratios: (a) #1 and #1', (b) #2 and #2', (c) #3 and #3', (d) #4 and #4', (e) #5 and #5', (f) #6 and #6', (g) #7 and #7', (h) #8 and #8', (i) #9 and #9'

POM images of PA56/512 copolymer

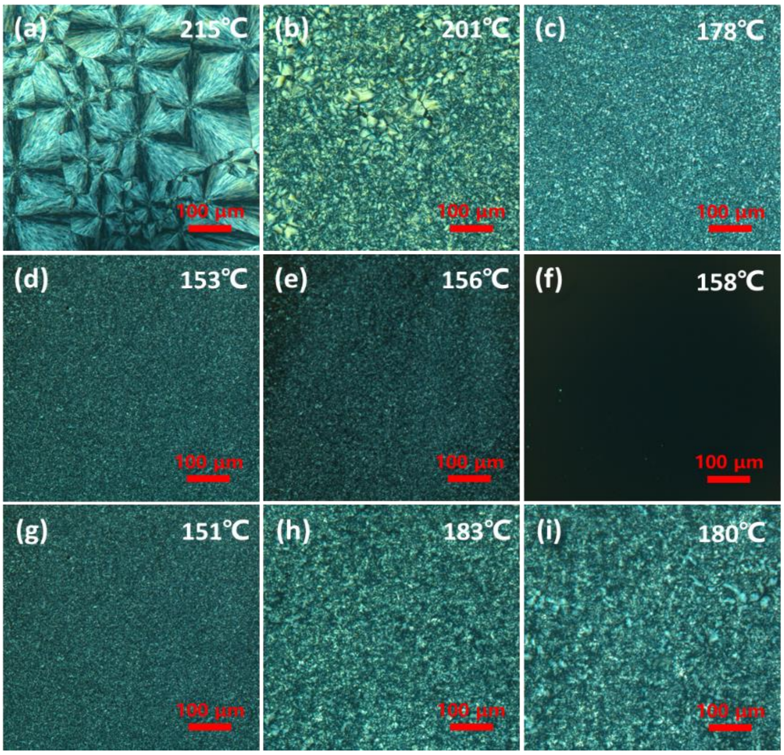

Fig. S3 POM images of PA56/512 before biaxial stretching: (a) #1, (b) #2, (c) #3, (d) #4, (e) #5, (f) #6, (g) #7, (h) #8, (i) #9

References

1. Xu, Y. H.; Zhao, C. Y.; Guo, Z. Y.; Dong, W. J.; Liu, X. C.; Guo, W. H. EPDM-g-MAH toughened bio-based polyamide 56 to prepare thermoplastic polyamide elastomer and the performance characterization. *Journal of Applied Polymer Science* **2022,** *139*, 52346.

2. Yang, K. J.; Liu, Y. L.; Zheng, Z. K.; Tang, Z. B.; Chen, X. D. Controlled polymerization and side reaction mechanism of bio-sourced pentanediamine-derived semi-aromatic copolyamides. *Polymer Chemistry* **2023,** *14*, 2390-2404.

3. Zheng, Y.; Pan, P. J. Crystallization of biodegradable and biobased polyesters: Polymorphism, cocrystallization, and structure-property relationship. *Progress in Polymer Science* **2020,** *109*, 101291.

4. Yu, Y.; Sang, L.; Wei, Z. Y.; Leng, X. F.; Li, Y. Unique isodimorphism and isomorphism behaviors of even-odd poly (hexamethylene dicarboxylate) aliphatic copolyesters. *Polymer* **2017,** *115*, 106-117.

5. Perez-Camargo, R. A.; Arandia, I.; Safari, M.; Cavallo, D.; Lotti, N.; Soccio, M.; Muller, A. J. Crystallization of isodimorphic aliphatic random copolyesters: Pseudo-eutectic behavior and double-crystalline materials. *European Polymer Journal* **2018,** *101*, 233-247.

6. Gan, D. S.; Liu, Y. J.; Hu, T. H.; Fan, S. H.; Liu, X. C.; Cui, L. N.; Yang, L.; Wu, Y. C.; Chen, L.; Mo, Z. X. The Investigation of Copolymer Composition Sequence on Non-Isothermal Crystallization Kinetics of Bio-Based Polyamide 56/512. *Polymers* **2023,** *15*, 2345.

7. Liu, L. C.; Lai, C. C.; Lu, M. T.; Wu, C. H.; Chen, C. M. Manufacture of biaxially-oriented polyamide 6 (BOPA6) films with high transparencies, mechanical performances, thermal resistance, and gas blocking capabilities. *Materials Science and Engineering B-Advanced Functional Solid-State Materials* **2020,** *259*, 114605.

8. Damari, S. P.; Cullari, L.; Nadiv, R.; Nir, Y.; Laredo, D.; Grunlan, J.; Regev, O. Graphene-induced enhancement of water vapor barrier in polymer nanocomposites. *Composites Part B-Engineering* **2018,** *134*, 218-224.
